# When You Have Lived in a Different Culture, Does Returning ‘Home’ Not Feel Like Home? Predictors of Psychological Readjustment to the Heritage Culture

**DOI:** 10.1371/journal.pone.0124393

**Published:** 2015-05-13

**Authors:** Laura Altweck, Tara C. Marshall

**Affiliations:** College of Health and Life Sciences, Brunel University, London, United Kingdom; Indian Institute of Science, INDIA

## Abstract

Many repatriates find it challenging to readjust to their heritage culture after spending a significant period of time abroad. Research on predictors of readjustment, however, remains limited. The present study in particular investigated the identification of third culture individuals (TCIs) – that is, individuals who spent their formative years outside of their heritage culture - with an abstract, third culture. Our findings demonstrated that TCIs’ identification with the third culture was empirically distinct from that of the heritage and host cultures. The present study further examined whether several variables – sojourner type (TCI vs. non-TCI), perceived conflict between heritage and host culture, perceived cultural distance, and cultural identification with heritage and other cultures – predicted psychological readjustment (stress, anxiety, depression and overall psychological readjustment). The results showed that strong heritage culture identification was associated with better psychological readjustment, whereas cultural conflict was generally associated with poorer readjustment. Furthermore, sojourner type significantly moderated the latter association, such that cultural conflict predicted the stress aspect of psychological readjustment for non-TCIs, but not for TCIs. As the present investigation is the first study to empirically establish identification with a ‘third culture’ we discuss implications for the literature on third culture individuals and psychological adjustment upon re-entry.

## Introduction

*“When I came home I expected everyone to have changed in the ways that I did*. *It’s shocking to find them doing the same things they did when I left*.*”* 20-year-old American female student who spent 11 months in England ([1], p. 415)

Once one has lived in another culture, discovering and embracing exotic music, food and traditions, one may return to one’s heritage culture only to find that ‘home’ is not what one remembered it to be. This feeling is widely reported in the re-entry literature, even suggesting that problems experienced during re-entry may be more serious than during adjustment to a foreign culture [1–6]. Several studies, theories and frameworks have proposed separate variables important to acculturation, adjustment, and the re-entry process. The main aim of the present study was to investigate the combination of variables that best predicts the psychological readjustment of sojourners on return to their heritage culture. We first review literature on acculturation and the psychological adjustment of migrants, then consider the psychological readjustment of expatriates.

### Acculturation

The classic definition of acculturation, devised by Redfield, Linton and Herskovits [7], conceptualises acculturation as the “phenomena which result when groups of individuals having different cultures come into continuous first-hand contact, with subsequent changes in the original culture patterns of either or both groups” (p. 149). Acculturation necessitates an exchange between two different cultures: the heritage culture, from which an individual or their parents are from, and a host or mainstream culture, which the acculturating individual has moved to.The ultimate goal of acculturation is adjustment, which denotes the relatively stable changes that take place in response to cultural transitions [8, 9]. Searle and Ward [10] first purported a duality to adjustment, namely psychological adjustment (mental and physical well-being) and socio-cultural adjustment (social skills in the mainstream/host culture). As the present investigation focuses on sojourners’ re-entry experiences, we assume that these individuals already possess the social skills that allow them to fit into the heritage culture, and therefore we only examined psychological readjustment.

Berry [11] conceptualised cultural identity along two independent dimensions, namely the degree of identification with heritage and host cultures, respectively. Berry [9] conceived of cultural identity as a person’s set of beliefs and attitudes that characterises how they see their relationship to their cultural group. Cultural identity has been largely studied from the perspective of heritage/ethnic and host culture identity [12–14]. Heritage culture identification refers to the sense of self, derived from its membership in the heritage culture—including beliefs about belongingness, feelings of commitment, and a sense of common values [13, 14]. Similarly, host culture identification denotes the sense of identity derived from feeling like a member of the mainstream culture [1, 13, 14]. Heritage and host culture identification have implications for psychological adjustment and readjustment.

#### Acculturation, Psychological Adjustment and Psychological Readjustment

The literature generally supports the notion that integrating one’s heritage and host cultures results in better psychological adjustment [8, 9], whether adjustment is conceptualised as low depression [15], positive self-image and few psychosomatic symptoms [16], work-related well-being (low burnout, affective organizational commitment, job satisfaction; [17]), self-esteem [18], mental health symptomatology [19], or better physical health [20, 21].

Substantially less research has been conducted on psychological adjustment on re-entry to the heritage culture. Sussman [22] devised a model of cultural identity change resulting from cross-cultural transitions. Identity changes where individuals become more similar to or different from the host culture were associated with lower clarity and stability of the self-concept, which may result in lower self-esteem, negative affective responses, and higher repatriation distress. Conversely, identity shifts that enabled sojourners to hold multiple cultural scripts simultaneously facilitated appropriate and effective interactions in many cultures. Sussman [22] hypothesised that the latter type of identity shift ameliorates re-entry distress and readjustment. Accordingly, Sussman’s [22] model proposes that strong heritage culture identification or integration of heritage and host culture identities allows for better readjustment. In support of this, Cox [23] found that home-favoured or integrated identity formation made the re-entry process significantly easier.

Thus, considering both the literature examining adjustment to another culture [8, 9, 17, 20, 24] as well as studies investigating readjustment to the heritage culture [22, 23, 25], we surmise that stronger identification with both the host and heritage cultures is associated with better psychological adjustment and readjustment.

## Perceived Cultural Distance

Berry [8] proposed that greater cultural distance between heritage and host cultures implicates greater cultural learning and shedding, potentially leading to migrants’ lesser socio-cultural adjustment. Yet there is ongoing debate regarding the relationship between cultural distance and psychological adjustment. In the original study on cultural distance, Babiker and colleagues [26] found a significant positive correlation between perceived cultural distance and anxiety among international students at a British university. Similarly, Redmond and Bunyi [27] found that students from Europe, the British Isles, and South America who attended an American university reported the least amount of stress, whereas students reporting the most stress were from the Mideast, China, Korea, and Japan. They concluded that students with backgrounds that shared more heritage, language, and culture with the USA reported the least amount of stress. Relatedly, Suanet and Van de Vijver [28] investigated perceived cultural distance in first year exchange students at Russian universities. They found that participants from Georgia, Uzbekistan and Ukraine, who reported smaller perceived cultural distance, experienced less stress and homesickness and socialized more with host nationals as compared to students that were not from former USSR states. They concluded that smaller perceived cultural distance aided people in adjusting better to the host country. Furthermore, Galchenko and Van de Vijver [29] found that participants who were lower in perceived cultural distance experienced better psychological adjustment (less stress and higher self-esteem).

However, other studies have found no significant association between perceived cultural distance and psychological state during acculturation [10, 15, 30]. For example, Nesdale and Mak [30] concluded that cultural distance was not predictive of psychological health among immigrants in Australia. However, one major limitation of their study was that the construct of cultural distance was measured via a single item requiring participants to indicate “how similar or different (they think their) ethnic background is to the Anglo-Australian culture” ([30], p. 28). Thus, it is questionable how well this represented participants’ perceived cultural distance. Furthermore, Searle and Ward [10] and Ward and Kennedy [15] found no association between perceived cultural distance and psychological adjustment. It is noteworthy that these studies modified the original, interview-based categories of Babiker and colleagues [26] into questionnaire-style items and asked participants to rate the categories along Likert-style scales. While this approach appears viable, neither of the studies presented the reliability of the adapted measure, and thus the accuracy of the results is questionable.

Overall, in light of the significant relationship found between perceived cultural distance and psychological adjustment in many studies [27–29, 31], we expected this association to translate to psychological readjustment.

## Cultural Conflict

Cultural conflict is an aspect of bicultural identity integration [32], which refers to the extent to which bicultural individuals view their two cultures as incompatible versus being in harmony [33]. Benet-Martìnez and colleagues [32] noted that although bicultural individuals identify with both host and heritage cultures, there are variations in the manner in which these two cultural systems are integrated [32]. While some biculturals are able to fluidly switch between cultural systems and see themselves as part of a “hyphenated culture,” others have difficulty forming a cohesive cultural identity and are very sensitive to tensions between the two cultural systems ([33], p. 1019). Further, individuals experiencing high cultural conflict may feel that they lack a cultural base—as though they are culturally confused or do not belong to a culture [34].

Chen, Benet-Martinez and Bond [34] investigated the association between cultural conflict and psychological adjustment in immigrants and sojourners living in Hong Kong. They found that lower perceived cultural conflict significantly predicted better psychological adjustment in immigrants from mainland China, but not in short-term sojourners. Moreover, Huynh, Devos and Smalarz [35] found that perceived conflict between cultural identities was positively correlated with self-rated depressive symptoms in immigrant American university students. These studies suggest that there is a negative relationship between cultural conflict and psychological adjustment, which, we expect to translate to psychological readjustment.

## Third Culture Individuals

Age is one of the most researched variables in the re-entry literature [36]. Indeed, Berry [8] proposed that if acculturation started at a young age, the process would generally run smoothly. He argued that acculturation into the parents’ culture may not be complete at a young age, and thus less culture shedding would need to occur after the child migrated to a new culture.

One particular type of sojourner that has received research attention is the Third Culture Kid. Because the majority of studies on the third culture experience do not focus on children, but rather examines these individuals as adults, the term Third Culture Individual (TCI) will be used instead. Pollock and van Reken ([6], p. 19) defined this type of individual as

a person who has spent a significant part of his or her developmental years outside the parents’ culture. The (TCI) builds relationships to all cultures, while not having ownership in any. Although elements from each culture are assimilated into the (TCI)’s life experience, the sense of belonging is in relationship to others of similar background.

The definition proposed by Pollock and van Reken [6] emphasises that TCIs identify less with a particular cultural group and more with people who have experienced and grown up with a similar third culture background. Cockburn [37] suggested that due to their exposure to different cultures, TCIs are more likely to hold a ‘worldview,’ paralleling Sussman’s [22, 25] notion that an individual who experienced global identity shifts is able to interact appropriately and effectively with people from many cultures. Furthermore, Moore and Barker [38] found that not only do some TCIs see themselves as excelling at shifting between cultural systems, but they also tended to blend their cultural identities into one overarching identity. Pollock and van Reken [6] defined this abstract ‘third culture’ as “created from the shared experiences and relationships with people from other cultures living the same lifestyle” (p. 191). While the importance of identification with the third culture has been highlighted in the TCI literature, to the best of our knowledge the present study is the first to attempt to empirically test it.

TCIs grow up learning to juggle different sets of cultural rules, and as a result their developmental process is different from people who grew up in a more homogenous environment [6]. While peers in the host and heritage culture have already internalized the norms, TCIs are still trying to figure out what the rules are. Transitioning between cultures may cause a disruption in identity development [39]; indeed, certain fundamental developmental tasks (e.g. identity achievement) that are usually mastered in adolescence in monocultural children may be delayed until the twenties and thirties in TCIs [6]. Thus it can be argued that when children move to a different culture, compared to adults, they are more likely to display lower heritage culture identification and ambiguous host culture identification.

In addition, the literature generally shows that individuals who moved to another culture at a younger age have more difficulty readjusting to their heritage culture [5, 23, 40]. At re-entry, younger sojourners reported feeling as if they “did not fit in” ([3]; p. 217), higher levels of depression and social difficulty [23], lower emotional stability [41], and elevated levels of anxiety, depression and stress alongside lower general psychological well-being [42]. Cox [23] suggested that younger sojourners had an advantage in learning the new culture during expatriation, which in turn made it more difficult to adapt during repatriation. Considering that stronger heritage culture identification is associated with better psychological adjustment [16, 24, 43], it is logical to surmise that TCIs’ lower heritage identification would mean that they would experience poorer psychological readjustment relative to non-TCIs at re-entry. Also, recall that individuals experiencing greater cultural conflict may feel that they lack a cultural base, as if they do not belong to a culture or are confused about their cultural identity [33, 44]. These characteristics are mirrored in the TCI literature [3, 38].

Overall, while a number of variables have been studied as predictors of psychological readjustment, to the best of our knowledge the present investigation is the first to examine cultural conflict, perceived cultural distance and cultural identification as predictors of psychological adjustment to the heritage culture at re-entry.

## Hypotheses

### Hypothesis 1

Identification with the third culture by TCIs is empirically distinct from identification with the heritage and host cultures.

### Hypothesis 2

Greater cultural conflict will (a) be reported by non-TCIs than TCIs, (b) be associated with weaker heritage culture and (c) with greater host culture identifications.

### Hypothesis 3

Compared to TCIs, non-TCIs will report (a) stronger heritage culture identification and (b) weaker host culture identification.

### Hypothesis 4

Greater third culture identification will be associated with greater cultural conflict.

### Hypothesis 5

(a) Stronger heritage culture identification, (b) weaker host and third culture identification, (c) lesser cultural conflict between heritage and host cultures and (d) weaker perceived cultural distance between heritage and host cultures will be associated with better psychological readjustment. Further (e) non-TCIs versus TCIs will report better psychological readjustment.

### Hypothesis 6

Sojourner type (TCI vs non-TCI) will moderate the associations of (a) cultural conflict, (b) host and (c) heritage culture identification with psychological readjustment. That is, the negative associations of cultural conflict and host culture identification with psychological readjustment will be stronger in TCIs, whereas the positive association of heritage culture identification with psychological readjustment will be stronger in non-TCIs.

## Method

### Ethics Statement

Ethical approval was obtained from the Brunel University Psychology Research Ethics Committee. Participants provided written informed consent at the beginning of the survey and all responses were confidential. Anonymized data is available on request from the first author.

### Participants

Two hundred and forty-eight participants who had lived in a culture other than their heritage culture and at some point returned to their heritage culture were recruited (70 males, 172 females). See Table 1 for participants” demographic information. The participant sample was divided into two sojourner types: TCIs and non-TCIs. The literature defines TCIs as individuals who have spent a significant part of their developmental years (birth until 18 years; [37]) outside of their heritage culture. There is great debate regarding the time period classed as ‘significant’; however, Cockburn ([37], p. 480) noted that “even a short stay abroad can have a significant impact upon the life of (a) child”. Therefore any participant who reported that they had lived abroad for a period of time was classified as a TCI if they had first moved to another culture at age 18 years or less (*N* = 97), or classified as an adult sojourner (non-TCI) if they first moved to another culture at 19 years or over (*N* = 132).

**Table 1:** Distribution of Demographic and Cross-Cultural Background Variables.

Variable	Mean / Frequency	Chi Square / *t*-test	Total Psychological Readjustment as DV
Age			
TCI	*M* = 29.2, *SD* = 10.9	*p* > .05	*p* > .05
non-TCI	*M* = 35.0, *SD* = 11.2		*N* = 233
Gender			
TCI	Male = 30, Female 72	*p* > .05	*p* > .05
non-TCI	Male = 37, Female = 100		*N* = 242
Reason for move			
TCI	Own choice = 88, other’s choice = 8	*p* > .05	*p* > .05
non-TCI	Own choice 40, other’s choice = 5		*N* = 133
No. cross-cultural moves			
TCI	*M* = 3.1, *SD* = 2.4	*t*(240) = 2.3,	*p* > .05
non-TCI	*M* = 2.4, *SD* = 1.8	*p* = .02	*N* = 233
Time abroad (years)			
TCI	*M* = 10.7, *SD* = 8.1	*t*(240) = 3.2,	*p* > .05
non-TCI	*M* = 7.4, *SD* = 8.1	*P* = .002	*N* = 233
Type of return			
TCI	Permanent = 84, Intermittent = 34	*p* > .05	*p* > .05
non-TCI	Permanent = 61, Intermittent = 24		*N* = 193
Length since return (years)			
TCI	*M* = 16.3, *SD* = 23.4	*p* > .05	*p* > .05
non-TCI	*M* = 13.7, *SD* = 20.3		*N* = 233

Age = age of participants at time of taking survey; Length since return = length since return from host to heritage culture

Participants originated from and lived in a diverse range of cultures, including Sri Lanka, Germany, Syria, Korea, Canada, Nicaragua, Czech Republic, India, South Africa, Bangladesh, USA, Sweden and Japan. Heritage culture was classified into three groups: English-speaking western (*N* = 103), European (*N* = 74) and Non-western (*N* = 64). As heritage culture group was a significant predictor of psychological readjustment, it was included as a control variable in all analyses. (Analyses revealed that non-Westerners reported significantly stronger heritage culture identification (*t*(136) = .87, *p* = .003) and greater anxiety (*t*(136) = 3.77, *p* < .001) than Europeans. Non-Westerners also reported stronger heritage culture identification (*t*(157) = 2.24, *p* = .003), greater anxiety (*t*(157) = 2.27, *p* = .02), and lesser cultural conflict (*t*(157) = -2.25, *p* = .03) than English-speaking Westerners.) As most participants experienced more than two cross-cultural transitions, often to very different cultures, we could not control for host culture in our analyses.

The cross-cultural experience of the present sample was diverse (see Table 1). Reasons for the cross-cultural transition (own choice—e.g. work/studies, other’s choice—e.g. parents job, accompany partner), number of cross-cultural transitions, amount of time abroad, type of return (permanent, intermittent) and length since return to heritage culture did not significantly predict any of the dependent variables (*p* > .05).

### Materials

#### Previous cross-cultural experience

Previous cross-cultural experience was assessed by asking participants to list their heritage and host cultures, and to indicate how long they had lived in each host culture, how many cross-cultural transitions they had experienced, how old they were when they first moved to another culture, reasons for moving abroad, reasons for returning and length of return to the heritage culture. Several participants had lived in and identified with a number of cultures; therefore, when responding to items that referred to the ‘host culture,’ we requested participants to respond according to the host culture that impacted them most. For all subsequent measures the instructions read, “Reminding yourself about when you returned to your heritage culture, please estimate how much you agree or disagree with the following statements”. To examine whether participants’ potential memory bias affected the current results we included length since return to heritage culture in all regression analyses, but because it did not significantly predict the dependent variables (*p* > .05) nor alter the significance of the main effects, it was removed from the models.

#### Perceived cultural distance

The Perceived Cultural Distance scale by Ait Ouarasse and Van de Vijver [45] was adapted by asking participants to indicate how much similarity they perceived between their host and heritage cultures in terms of 22 characteristics (e.g. *climate*, *religion*, *relation to family*, *social contacts*, *food*). The items were assessed on a 7-point Likert scale ranging from 1 (Very different) to 7 (Very similar). All items were reverse-coded so that higher scores indicated greater perceived cultural distance. The reliability for this scale was very high (α = .94).

#### Cultural conflict

The cultural conflict subscale of the Bicultural Identity Integration Scale (BIIS-1; [33]) measures perceived conflict between two cultural identities (in this case heritage and host culture identities). This subscale consists of four items that are assessed on a 5-point Likert scale ranging from 1 (Strongly agree) to 5 (Strongly disagree). Sample items include, “I feel like someone moving between two cultures” and “I am conflicted between the two cultures in ways of doing things”. Higher scores indicated greater conflict in heritage and host culture identities. The subscale was reliable (α = .79).

### Cultural identification

Cultural identification was measured with the Vancouver Index of Acculturation (VIA; [14]). The original measure is a bi-dimensional measure of identification with host and heritage cultures; however, as TCIs tend to endorse an abstract, third culture [6], another dimension was added to assess the identification of TCIs with the third culture. Participants who did not identify as TCIs were asked to indicate ‘N/A’ for the third culture items.

Each subscale consisted of 10 items to which participants were asked to indicate the extent to which they agreed. The subscales were identical except for reference group (i.e., *heritage*, *host* and *third culture*). Sample items include, “I am comfortable working with people from…” and “I often behave in ways that are typical of *…*” (*my heritage culture / the host culture / the third culture*). Items were rated on a 9-point Likert scale ranging from 1 (Strongly agree) to 9 (Strongly disagree) such that greater values reflected greater identification with that culture. Reliabilities for all three subscales were high; Cronbach’s alphas were. 90,. 86 and. 92 for heritage, host, and third culture subscales, respectively.

#### Psychological readjustment

The Depression Anxiety Stress Scale (DASS-21; [46]) was used to measure the psychological adjustment of participants at the point when they returned to their heritage culture. The DASS-21 is a three-dimensional measure that assesses depression, anxiety and stress, with each subscale consisting of seven items. Sample items include, “I found myself getting agitated” (stress subscale), “I felt that life was meaningless” (depression subscale), and “I felt scared without any good reason” (anxiety subscale). Participants were asked to rate items on a 5-point Likert-scale, ranging from 1 (Strongly disagree) to 5 (Strongly agree), such that higher scores indicated greater stress, depression and anxiety. The reliability of the subscales was high; Cronbach’s alphas were. 89,. 92 and. 90 for the stress, depression, and anxiety scales, respectively.

Because the subscales were highly correlated (*r*s > .60), we created a composite variable reflecting total psychological readjustment. For this variable only, the items were reverse-coded so that higher scores reflected lower stress, depression, and anxiety (i.e. better psychological readjustment); *z*-scores were then computed for each subscale and summed to create a composite score. The reliability for this composite variable was high (α = .96).

### Procedure

The study was conducted online through a survey-building website. A hyperlink to the survey was distributed through the university’s intranet site and social networking sites such as Facebook, Twitter and Pinterest. Participants were invited to complete a survey on readjustment to one’s heritage culture. IP addresses were inspected to ensure there were no multiple entries. All materials were in English only.

## Results

### Hypothesis 1: Third Culture identification

To test Hypothesis 1—whether third culture identification by TCIs was empirically distinct from identification with the heritage and host cultures—a principal component analysis (PCA) with a varimax rotation was conducted. Initial analysis revealed that six factors held eigenvalues above Kaiser’s criterion of 1, but inspection of the scree plot revealed only three clear factors. Sampling adequacy for an analysis was validated through the Kaiser-Meyer-Olkin measure, KMO = .83, and all KMO values for individual items were greater than. 70. Bartlett’s test of sphericity confirmed the presence of substantial correlations between the items, χ^2^ (435) = 4969.92, *p* < .001. The three extracted factors accounted for 55% of the total variance. Table 2 shows the factor loadings of the items after rotation. Items were retained if they had loadings above. 40, except if they showed considerable cross-loading. All items of the heritage and third culture subscales loaded onto their respective factor and displayed loadings of. 50 or greater. Factor 3 reflects the majority of the host culture identification subscale, yet some of the items did not display loadings greater than. 40. This includes the items ‘I would be willing to marry a person from the host culture’, ‘I am comfortable working with people from the host culture’ and ‘I am interested in having friends from the host culture’. One explanation for these non-significant loadings is that the majority of participants had lived in more than one host culture. Even though participants were asked to respond according to the host culture that impacted them most, it is possible that these items were difficult to answer. The non-loaded items were removed from the host culture identification scale and the derived subscales were used in all following analyses.

**Table 2:** Factor loadings for the VIA (significant factor loadings are bolded).

	Rotated Factor Loadings		
	Heritage CI	Host CI	Third CI
.	**.545**		
I would be willing to marry a person from my heritage culture.	**.635**		
I enjoy social activities with people from my heritage culture.	**.750**		
I am comfortable working with people my heritage culture.	**.721**		
I enjoy entertainment (e.g. movies, music) from my heritage culture.	**.732**		
I often behave in ways that are typical of my heritage culture.	**.808**		
It is important for me to maintain or develop cultural practices of my heritage culture.	**.624**		
I believe in the values of my heritage culture.	**.787**		
I enjoy the jokes and humour my heritage culture.	**.777**		
I am interested in having friends from my heritage culture.	**.746**		
I often participate in traditions of the host culture.		**.580**	
I would be willing to marry a person from the host culture.			
I enjoy social activities with people from the host culture.		**.440**	
I am comfortable working with people the host culture.		.292	
I enjoy entertainment (e.g. movies, music) from the host culture.		**.602**	
I often behave in ways that are typical of the host culture.		**.699**	
It is important for me to maintain or develop cultural practices of the host culture.		**.787**	
I believe in the values of the host culture.		**.757**	
I enjoy the jokes and humour the host culture.		**.635**	
I am interested in having friends from the host culture.			
I often participate in traditions of the third culture.			**.536**
I would be willing to marry a person from the third culture.			**.660**
I enjoy social activities with people from the third culture.			**.904**
I am comfortable working with people the third culture.			**.872**
I enjoy entertainment (e.g. movies, music) from the third culture.			**.773**
I often behave in ways that are typical of the third culture.			**.677**
It is important for me to maintain or develop cultural practices of the third culture.		**.464**	**.528**
I believe in the values of the third culture.			**.767**
I enjoy the jokes and humour the third culture.			**.816**
I am interested in having friends from the third culture.			**.873**
Eigenvalues	4.18	3.67	7.70
% of total variance explained	12.22	10.75	32.06
α	.90	.86 *(*.*84)*	.92
Number of items	10	10 *(7)*	10

Overall, Hypothesis 1 was supported: third culture identification was found to be empirically distinct from identification with heritage and host cultures, respectively.

### Hypothesis 2, 3 and 4: Cultural Conflict, Heritage, Host and Third Culture Identification

Means and correlations between variables are reported in Tables 3 and 4. The Kolmogorov-Smirnov (*p* < .04) and Shapiro-Wilk (*p* < .001) tests were significant for the main independent and dependent variables; however, this is expected due to our large sample size. To test hypothesis 2a —that cultural conflict will be reported less by TCIs than non-TCIs—we conducted a hierarchical regression analysis with cultural conflict as the dependent variable. The categorical predictors were coded as follows: gender (male = 1, female = -1), heritage culture group (contrast 1: non-Western = 1, European = 0, English-speaking Western = -1; contrast 2: non-Western = 1, European = -1, English-speaking Western = 0), and sojourner type (TCI = 1, non-TCI = -1). Results are reported in Table 5. Only age significantly predicted cultural conflict, indicating that older participants reported greater conflict between heritage and host cultural identities. Sojourner type trended towards significance; the negative association indicated that non-TCIs tended to report greater cultural conflict than TCIs, lending some support to Hypothesis 2a.

**Table 3:** Correlations between key variables (total sample under the diagonal and TCI sample only over the diagonal).

		1	2	3	4	5	6	7	8	9
1.	Cultural conflict		-.03	-.09	.08	.08	.06	.11	-.07	.09
2.	PCD	-.10		.08†	.17	.13	.08	.14	-.07	.01
3.	Heritage culture identification	-.22***	.11		-.31**	-.33***	-.29**	-.26*	.29***	-.33***
4.	Host culture identification	.04	.17**	-.19**		.04	.06	.05	-.02	-.33***
5.	Stress	.22***	.13*	-.28***	.01		.82***	.81***	-.88***	-.15
6.	Depression	.15*	.02	-.28***	.04	.76***		.82***	-.92***	-.08
7.	Anxiety	.22***	.12	-.27***	.06	.75***	-.75***		-.92***	-.17
8.	Total Readjustment	-.21***	-.09	.30***	-.04	.83***	-.86***	-.90***		.15
9.	Third culture identification	-	-	-	-	-	-	-	-	

†*p* < .10

*p < .05

**p < .005

***p < .001.

**Table 4:** Key variable means and associations with sojourner type.

	Means (Standard Deviations)		
Variable	TCI	Non-TCI	*t*-tests
Perceived cultural distance	53.60 (19.60)	62.39 (25.36)	*t*(240) = -2.94, *p* = .004
Cultural conflict	11.46 (4.01)	9.77 (3.21)	*t*(240) = -3.02, *p* < .001
Heritage culture identification	28.87 (16.70)	25.46 (13.00)	*t*(240) = 1.79, *p* = .08
Host culture identification	22.11 (10.65)	25.61 (9.87)	*t*(240) = -2.64, *p* = .009
Third culture identification	23.58 (17.14)	-	-
Stress	17.50 (7.45)	17.51 (6.54)	*p* > .05
Depression	16.05 (7.95)	15.34 (6.83)	*p* > .05
Anxiety	14.77 (6.81)	12.85 (5.58)	*t*(239) = 2.41, *p* = .02

**Table 5:** Regression models with cultural conflict and cultural identification (heritage, host, and third, respectively) as the dependent variables.

			Cultural Identification		
		Cultural Conflict	Heritage	Host	Third
Model		β	β	β	β
Step 1	Age	.15**	-.03	.05	-.14
	Gender	-.02	-.05	-.07	-.10
	Heritage culture group				
	Contrast 1	-.03	.15*	-.01	-.05
	Contrast 2	-.17	-.02	-.13†	.10
	R^2^	.06***	.03	.02	.03
Step 2	Sojourner type	-.13†	.07	-.14*	-
	Cultural Conflict	-	-.13	.07	-.04
	∆R^2^	.01†	.02†	.05*	.008
*N*		235	235	235	98

†*p* < .10

*p < .05

**p < .01

***p < .006; Age = age of participants at time of taking survey

Next we investigated the second part of Hypothesis 2—that (b) greater cultural conflict will be associated with weaker heritage cultural identification and (c) with greater host culture identification—as well as Hypothesis 3—that non-TCIs will report (a) stronger heritage culture identification and (b) weaker host culture identification. We conducted a hierarchical regression analysis with heritage and host culture identification as the dependent variables respectively (see Table 5).

In relation to heritage culture identification, heritage culture group contrast 1 was significant, with non-Westerners reporting stronger heritage culture identification than English-speaking Westerners. Sojourner type was non-significant—refuting H3a —but cultural conflict was significantly negatively associated with heritage culture identification. This supports Hypothesis 2b, indicating that participants who reported greater cultural conflict identified less strongly with their heritage culture.

In relation to host culture identification, only sojourner type was found significant in a negative direction. These findings contradict Hypothesis 2c, but lend support to hypothesis 3b, confirming that TCIs reported weaker host culture identification than non-TCIs.

To examine Hypothesis 4—stronger third culture identification will be associated with greater cultural conflict—we conducted a regression analysis on the TCI sample only with third culture identification as the dependent variable. As reported in Table 5, none of the control nor predictor variables were significant, refuting Hypothesis 4.

### Hypotheses 5 and 6: Psychological Readjustment

Next we examined Hypothesis 5—whether better psychological readjustment at re-entry will be (a) associated with stronger heritage culture identification, (b) weaker host and third culture identification, (c) lesser cultural conflict between heritage and host cultures, (d) weaker perceived cultural distance between heritage and host cultures as well as whether(e) non-TCIs compared to TCIs will report better psychological readjustment—and Hypothesis 6—sojourner type will moderate the associations of cultural conflict, host and heritage culture identification with psychological readjustment. We conducted four hierarchical regression models with total psychological readjustment and each subscale (stress, depression, and anxiety) as dependent variables respectively. Control variables were entered in the first step, and sojourner type, cultural conflict, perceived cultural distance, heritage culture identification, and host culture identification were entered in the second step. Interactions of sojourner type with cultural conflict, heritage identification, and host identification were entered in the third step.

As reported in Table 6, sojourner type did not significantly predict any of the dependent variables, disconfirming Hypothesis 5e. In support of Hypothesis 5a, the results showed that greater heritage culture identification was signficantly associated with lower stress, anxiety, and depression and with greater overall psychological readjustment above and beyond any control variables. These findings underline the importance of heritage culture identification for enhanced psychological readjustment. Furthermore, cultural conflict was significantly associated with greater stress and anxiety, and lower total psychological readjustment above and beyond the control variables—partially supporting Hypothesis 5c.

**Table 6:** Regression models with stress, depression, anxiety, and total psychological readjustment as dependent variables.

Model		Stress	Depression	Anxiety	Total PsychologicalReadjustment
		β	β	β	β
Step 1	Age	.14*	.16**	.13*	-.10
	Gender	-.03	-.04	-.03	.03
	Heritage culture contrasts				
	Contrast 1	-.10	-.07	-.01	.00
	Contrast 2	-.15*	-.13†	-.25***	.16*
	R^2^	.04†	.04†	.08***	.04†
Step 2	Sojourner type	.12†	.05	.02	-.00
	Cultural conflict	.15**	.09	.15**	-.15*
	Perceived cultural distance	.12†	.00	.08	-.04
	Heritage culture identification	-.28***	-.29***	-.23***	.28***
	Host culture identification	.04	.05	.05	-.06
	∆R^2^	.13***	.09***	.10***	.11***
Step 3	Host culture identification × Sojourner type	.08	.11	.01	-.01
	Heritage culture identification × Sojourner type	-.07	-.03	-.01	.08
	Cultural conflict × Sojourner type	-.58*	-.26	-.40	.53†
	∆R^2^	.02	.01	.007	.01
*N*		235	235	235	235

†*p* < .10

*p < .05

**p < .03

***p < .001; Age = age of participants at time of taking survey

Further hierarchical regression models were conducted with just the TCI sample to test Hypothesis 5b —that third culture identification negatively predicts psychological readjustment. The same control, predictor and outcome variables that were entered in the previous regression analyses were included again here, and third culture identification was entered as an additional predictor variable. None of the analyses revealed third culture identification as a significant predictor (*p* > .05; *N* = 98), which refuted Hypothesis 5b. Otherwise, the findings were similar to the previous results: none of the control variables were significant and of the predictor variables, only heritage culture identification showed a significant association above and beyond all control variables (stress: β = -.34, *p* = .002; depression: β = -.33, *p* = .003; anxiety: β = -.22, *p* < .05; psychological readjustment: β = .30, *p* = .008).

Finally, cultural conflict only significantly interacted with sojourner type when predicting stress, lending support to H6a, but refuting H6b and H6c. Simple slope analysis revealed that cultural conflict significantly predicted greater stress at re-entry for non-TCIs (β = .26, *p* = .002), but not for TCIs (*p* > .05).

## Discussion

The present study investigated the predictors of psychological adjustment upon re-entry to one’s heritage culture. The results indicated that heritage culture identification and cultural conflict were the most important predictors of psychological readjustment. We also found that endorsement of third culture identification was empirically distinct from heritage and host culture identification.

### Third Culture Identification

In this study, TCIs’ identification with the third culture—such as its humour, traditions, friends and social activities—was significantly different from identification with heritage and host cultures. Although this third culture has been an important aspect of the TCI literature [6], to the best of our knowledge the present paper is the first to provide empirical support for identification with this abstract, third culture. This is an important finding as it lends credence to frameworks representing the influence of the third culture on TCIs’ cognition, behaviours and development [3, 6].

### Cultural identification

Of the three types of cultural identification investigated in the present study, heritage culture identification was the strongest predictor of psychological readjustment. Most studies have found that strong host culture identification or integration of heritage and host cultures optimally facilitate psychological adjustment to a new culture [8, 9, 22, 23, 25]. Nguyen and colleagues [47], who found that host culture involvement was most important when acculturating to another culture, concluded that maintaining one’s heritage culture may be stressful and hinder adaptation to the host culture. A parallel may be drawn to readjustment. Although the present study did not find third and host cultures to hinder psychological readjustment, host or third culture identities may simply be insignificant when readapting to the heritage culture. Furthermore, strong heritage culture identification may be associated with relevant information and skills (such as language ability or social skills), which would benefit readjustment. In conclusion, sojourners who strongly identify with the culture to which they are acculturating—whether it is the host or heritage culture—will experience better psychological adjustment.

The results further showed that TCIs identified less strongly with the host culture than non-TCIs. Pollock and van Reken [6] suggested that because TCIs grew up in another culture, they would internalise these host cultural rules and norms to a greater extent than sojourners who lived in another culture as adults; however, this was not confirmed in the present study. While the experience of growing up in another culture may teach TCIs to learn to switch between and adapt to cultural systems, this appears to be distinct from identifying strongly with particular host cultures. Instead, we found that TCIs identified more strongly with the abstract third culture, than a specific host culture.

Furthermore, the present findings showed that sojourner type was not significantly related to heritage culture identification. On the one hand, this may be due to TCIs visiting their heritage culture throughout their formative years and their parents acting as the main conduit through which children learn about their heritage culture. As a result children would learn about their heritage culture more or less equally whether they live in their heritage culture or not. On the other hand, the third culture experience enables TCIs to quickly learn cultural rules and norms [22, 37, 38]. This would allow TCIs to easily grasp aspects of their heritage culture, therefore making TCIs’ heritage culture identification indistinguishable to non-TCIs’ identification.

### Cultural Conflict

Individuals who reported greater conflict between heritage and host culture identities experienced greater stress and anxiety and worse overall psychological readjustment. Previous research has also found that cultural conflict is associated with a neurotic disposition and psychological distress [33, 34]. Individuals experiencing greater cultural conflict may be more vigilant and looking for clues to identify which cultural system to draw upon in a particular situation. Therefore it is possible that cultural conflict was non-significantly related to depression because depression generally has an underlying apathetic quality to it.

Furthermore, cultural conflict was significantly moderated by sojourner type when predicting stress. Indeed, cultural conflict showed a significant positive association with stress in non-TCIs, but not in the TCI sample. TCIs have been shown to be flexible in alternating between different cultural systems and therefore cultural conflict may not depend on identification with a specific cultural system [6, 38]. For non-TCIs, however, the heritage culture may have become a more ingrained cultural reference point, increasing the confusion between cultural systems and in turn yielding greater levels of stress. One explanation for why the moderation occurred only for the stress component of psychological readjustment is that cultural conflict is associated with general stress rather than to more specific psychopathologies like depression or anxiety; in any case, this distinction warrants further research.

### Perceived Cultural Distance

The present results showed that perceived cultural distance only trended towards significance as a predictor of stress, in contrast to previous studies [29, 30]. These studies examined sojourners’ experiences of acculturation to another culture, where perceived cultural distance was found to be an important variable [27, 28]. The discrepancy in results may be due to our focus on re-entry experiences as opposed to acculturation to another culture. It is reasonable to assume that individuals already possess solid social knowledge and competencies about their heritage culture when they return, therefore the individuals’ perceived difference in host and heritage culture may be inconsequential in relation to the re-entry experience.

### Limitations and future directions

The present study had several limitations, but they did not detract from the overall implications. First, the sample was diverse both in terms of heritage and host cultures. On the one hand, participants were divided into broad heritage culture categories, and we controlled for this variable in our analyses. On the other hand, several participants had lived in more than one host culture, and although the survey required participants to focus on the most influential host culture, it is possible that this multitude of host cultures may have detracted from the strength of the variable measuring host culture identification. To ensure greater control, future studies would do well to examine participants from specific heritage culture groups who have lived in just one host culture.

A second limitation of this study is the retrospective approach. Participants’ memories of their thoughts and feelings when re-entering their heritage culture may have eroded over time. While the associations between time since re-entry and the dependent variables were non-significant, and including time since re-entry in the regression models did not alter the main patter of results, future research should examine the psychological readjustment of individuals who have recently returned to their heritage culture.

Third, we found relatively low R-square values in the present regression analyses. Although it is unwise to place too much emphasis on this—because it would be necessary to enter predictor variables in a regression analysis that are very similar to the outcome variable to yield high R square values [48]—it nonetheless shows that there are further important determinants of the studied outcome variables. Indeed the associations of heritage culture group with psychological readjustment were unexpected and deserve greater attention in subsequent research. Non-westerners reported significantly stronger heritage culture identification and lesser anxiety than Europeans. Furthermore, non-Westerners also reported stronger heritage culture identification, greater anxiety and lesser cultural conflict than English-speaking Westerners. One possible explanation is that due to globalisation, non-Westerners are increasingly exposed to Western cultures. These results underline the importance of heritage culture in relation to acculturation and psychological readjustment. In the same fashion, Gullahorn and Gullahorn [4] and Martin [5] proposed that location of sojourn would be an important factor affecting re-entry. Arguably, culture plays an important part in psychological readjustment and therefore heritage and host culture as predictor variables should feature more in future acculturation, adjustment and re-entry research.

### Conclusion

In the present study, heritage culture identification and cultural conflict emerged as the best predictors of psychological readjustment: stronger heritage culture identification facilitated whereas greater cultural conflict impeded psychological readjustment. Furthermore, sojourner type significantly moderated cultural conflict in relation to stress, with cultural conflict showing a significant positive association with stress in non-TCIs but not in TCIs. Moreover, we empirically established that endorsement of the third culture was distinct from heritage and host culture identification. Future research would do well to determine the predictors of third culture identification as well as its implications. We also need to further investigate the predictors of psychological adjustment on re-entry to better understand sojourners’ feelings of returning to their heritage culture and yet not quite feeling at ‘home’.

## Supporting Information

S1 SPSS (ZIP)
